# Determinants and Consequences of Long Impact of COVID-19: A National Cross-Sectional Study in Saudi Arabia

**DOI:** 10.3390/jcm15155902

**Published:** 2026-07-28

**Authors:** Abdulaziz Alshomrani, Anum S. Hussaini, Elhadi Miskeen

**Affiliations:** 1 Department of Internal Medicine, College of Medicine, University of Bisha, Bisha P.O. Box 67976, Saudi Arabia; 2 Department of Global Health and Population, Harvard T.H. Chan School of Public Health, Boston, MA 02115, USA; anum.shussaini@gmail.com; 3 Department of Obstetrics and Gynecology, College of Medicine, University of Bisha, Bisha P.O. Box 67976, Saudi Arabia

**Keywords:** COVID-19 sequelae, post-acute COVID-19, population health, chronic fatigue, cognitive dysfunction

## Abstract

**Background:** The post-acute consequences of prolonged Coronavirus Disease 2019 (COVID-19) infection are a major public health problem characterized by long-lasting, multisystem manifestations. There is limited evidence from Saudi Arabia, particularly from nationally representative studies using standard case definitions. **Objective:** This study aimed to estimate the prevalence of Long COVID, characterize its symptoms, identify risk factors, and assess disease burden and healthcare utilization among Saudi adults. **Methods:** Between June 2023 and December 2024, we conducted a national cross-sectional mixed-methods study among 1790 adults with PCR-confirmed COVID-19 infection in all 13 regions of Saudi Arabia. Long COVID was defined using the WHO consensus case definition (symptoms persisting ≥ 3 months post-infection not attributable to alternative diagnoses). **Results:** The prevalence of Long COVID was 32.0% (95% CI 29.8–34.3). The most common symptoms were fatigue (68%), dyspnea (45%), and cognitive impairment (39%). Independent predictors included female sex (adjusted odds ratio [aOR] 1.8, 95% CI 1.4–2.3), moderate-to-severe acute infection (aOR 2.5, 95% CI 2.0–3.1), incomplete vaccination (aOR 1.6, 95% CI 1.2–2.1), metabolic disorders (aOR 1.6, 95% CI 1.2–2.1), and obesity (aOR 1.5, 95% CI 1.2–1.9). Among Long COVID patients, 41% required specialty care and 12% reported work disability. **Conclusions:** Long COVID affects approximately one-third of COVID-19 survivors in Saudi Arabia, imposing a significant healthcare and socioeconomic burden. These findings highlight the need for swift action and policy implementation within the health care system to mitigate long-term consequences.

## 1. Introduction

Since the onset of the COVID-19 pandemic, we have transitioned from an acute global emergency to a chronic public health crisis affecting millions worldwide [1]. Long-term impact (defined by the WHO as symptoms persisting for more than 3 months) also affects health systems, workforce productivity, and quality of life, which emphasizes its importance in regional health planning [2,3].

It is clear from global epidemiological data that Long COVID has become a major and persistent burden seen across all populations. The worldwide prevalence is approximately 36% according to recent systematic reviews and meta-analyses. But that is not uniform, ranging from 20% for certain Asian populations to >50% in South American cohorts [1,4]. Most importantly, prevalence rates are consistent across long follow-up periods at 2 years post-infection) [1]. Data on disease prevalence in the Middle East and North Africa are still scarce, but initial evidence suggests rates similar to those in other countries. In studies conducted in Saudi Arabia, the prevalence of coronavirus over time varied between 28% and 51%, with considerable heterogeneity explained by differences in case definition, follow-up time, and study population [2,5,6].

Long-term coronavirus is a multi-organ disease characterized by symptoms appearing in a range of organs and organ systems. The fatigue is the most common symptom, followed by shortness of breath (18–45%) and cognitive impairment (18–39%), affecting up to 68% of survivors [1,4,7]. Indeed, neurological sequelae (memory impairment, impaired concentration, and headache) similarly impact close to 16–19% of Long COVID patients who continue to show symptoms on follow-up at one year [8]. Cardiovascular symptoms (chest pain and palpitations) and psychological manifestations (anxiety and depression) also highlight this disease’s multi-systemic presentation [9,10].

Long COVID poses a considerable and complex healthcare and socioeconomic burden. Around 40% of patients with Long COVID require care from specialists, adding significantly to the burden already placed on healthcare systems by demands associated with the pandemic [2,11]. Work disability is prevalent in 12–15% of survivors with serious consequences for labor force participation and economic productivity [12]. The growing burden on health systems in Saudi Arabia has led to this challenge being recognized at the national level and to the exploration of the simultaneous establishment of coordinated care pathways post-COVID [2]. However, qualitative evidence points to persistent challenges, including delays in diagnosis and limited awareness among health care providers [13,14].

While the global literature on long-term coronavirus is growing rapidly, significant gaps in knowledge regarding its epidemiology and determinants remain in the MENA region. However, most existing studies in Saudi Arabia were central or regional studies, with limited generalizability [5,6,15].

The purpose of the study was to estimate the long-term spread of the coronavirus, characterize its symptoms, identify risk factors, and assess the burden of disease and healthcare utilization of Saudi adults. Employing a high-fidelity cross-sectional study design with bimodal data capture and clinically endogenous WHO-recommended case definitions, this study aims to produce strong evidence to inform clinical treatment strategies and public health policy decisions for resource allocation in Long COVID care delivery in Saudi Arabia, and more broadly across the MENA region.

## 2. Methodology

**Study design and reporting:** This was a community-based, national cross-sectional study with a concurrent mixed methods design, conducted across all 13 administrative regions of Saudi Arabia over 18 months (June 2023–August 2024–December 2024), the June 2023–August 2024 period was devoted exclusively to pre-approval preparatory activities (protocol registration, tool development, and administrative arrangements), not to data collection, the official study period as beginning strictly from the date of IRB approval (21 August 2024). The study is reported according to the STROBE statement. For the quantitative component, data on Long COVID prevalence, risk factors, and healthcare utilization were collected cross-sectionally. For the qualitative component, a subset of participants was invited to provide open-ended responses regarding their lived experiences with persistent symptoms. Symptom trajectories were assessed retrospectively: participants were asked to recall the presence, duration, and resolution of symptoms at each time point during a single cross-sectional interview or survey. No prospective longitudinal follow-up was conducted.

**Operational definition of Long COVID:** Long COVID was operationalized according to the 2021 WHO consensus case definition of symptoms ≥ 3 months following PCR-confirmed COVID-19 infection persisting for a minimum of 2 months and not explained by an alternative diagnosis. The symptom domains were fatigue, dyspnea, and cognitive impairment [16].

**Recruitment:** We employed a three-step stratified random sampling design, where: (1) we first divided the country into strata by administrative region (a total of 13). For each region, we distributed the target sample size in accordance with the proportion of adults living in that region. In the second stage, we randomly sampled primary sampling units (regions) within each region and randomly selected eligible individuals within each region.

**Figure 1:**
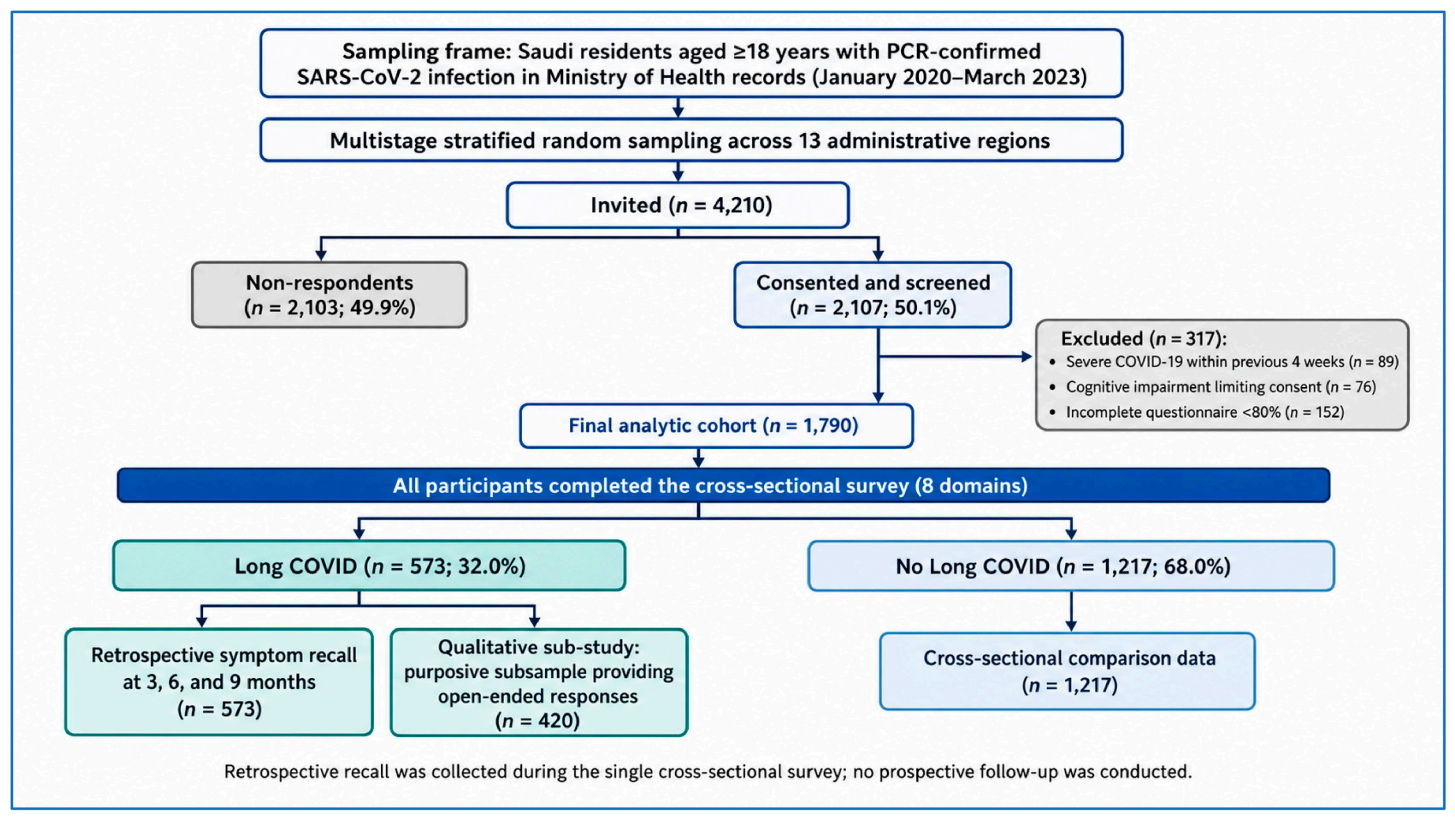
Participant Flow Diagram for the National Cross-Sectional Study of Long COVID in Saudi Arabia (*n* = 1790).

Figure 1 shows the number of individuals invited, screened, enrolled, and included in the final analytic sample, with reasons for exclusion. All three study components (cross-sectional survey, retrospective recall, and qualitative sub-study) were drawn from this single cohort.

The draft questionnaire was pilot-tested with approximately 50 subjects, who were excluded from the analysis. Pilot data were used to clarify wording, the order of items, and the feasibility of the mode (telephone or online). The wording of the symptom and functional-status questions was harmonized when necessary to ensure semantic equivalence between the Arabic and English versions and to minimize misclassification due to language differences.

**Quality of the Data Collection:**

Data was collected through a validated questionnaire as an online survey. This pilot-tested tool (*n* = 50, excluded from final analysis) includes eight domains: sociodemographic, acute COVID-19 history (signs/symptoms and hospitalization need), vaccination uptake, persistent symptom frequency, comorbidities, healthcare utilization, functional status, and socioeconomic effects. The full questionnaires are provided as Supplementary File S1.

**Statistical Analysis**

The STATA BE version 19; (Stata Corp LLC, College Station, TX, USA), (2025). Software was used for the data analyses. Continuous variables were expressed as means (IQRs). We did convert categorical variables into numbers. We examined the associations between items and Long COVID using bivariate methods (chi-square tests, *t*-tests, or Mann-Whitney U tests).

Less than 5% of variables contained missing data, and there were no significant differences in Long COVID status for those that had missing data (Little’s MCAR test, *p* = 0.34). The complete-case analyses fitted the main multivariable models, while both sensitivity analyses using multiple imputation yielded similar results that provided evidence of robustness.

Similar results were also found when using multivariable logistic regression, which identified independent predictors. The model included age, sex, and prior comorbidities forced in; other candidate predictors were selected using backward stepwise elimination (entry *p* < 0.10, retention *p* < 0.05). Due to the limited Saudi data, stepwise regression was exploratory, and conclusions were drawn from those of the fully adjusted model. Multicollinearity was evaluated (mean VIF = 1.42, range 1.1–2.3). The AUC (0.78) and the Hosmer–Lemeshow test (*p* = 0.42) were used to assess the model’s performance.

We conducted sensitivity analyses with alternative definitions of acute severity and vaccination status. Demographic and clinical strata were used to perform subgroup analyses to examine heterogeneity.

**Qualitative Data**

Qualitative open-text responses (*n* = 420) were collected from a subsample selected through predefined purposive sampling of the main cohort. The interview consisted of a wide variety of free-text questions covering topics such as the burden of symptoms, issues with health service use, and social and coping challenges. Two bilingual researchers thematically analyzed the responses. Common qualitative themes identified through deductive analysis were mapped to quantitative findings using a framework for inductive thematic synthesis. Integration took place in the knowledge-to-action step, at the interpret stage, through constructing a joint display, where each theme was related to its approximate quantitative variable. Validity was also bolstered through subsample participant verification.

## 3. Results

The prevalence of Long COVID was 32.0% (95% CI 29.8–34.3). Fatigue was the most common symptom (68%), followed by dyspnea (45%) and cognitive impairment (39%).

The main risk factors are female gender, moderate to severe acute infection, and underlying metabolic disorders. Longitudinal data showed gradual improvement in symptoms, but 15% of patients developed permanent disability after six months.

The demographic, clinical, and socioeconomic data, along with their association with Long COVID prevalence, are presented in Table 1. Out of those with PCR-confirmed COVID-19 (*n* = 1792), 32.0% (*n* = 573) had Long COVID symptoms extending three or more months post-infection. The Long COVID group differed significantly from the others on most of the variables were older (mean age 47.5 versus 44.1 years, *p* < 0.001), were more likely to be female (62.0% versus 47.3%, *p* < 0.001), and had a higher proportion of Saudi nationals (73.5 versus 68.4%, *p* = 0.03). Long COVID cases were more likely to reside in rural areas (22.9% vs. 18.6%, *p* = 0.02) and to be unemployed (40.3% vs. 29.4%, *p* < 0.001). However, there was a strong between Long COVID and clinically moderate-to-severe acute infection (52.0% vs. 27.0%, *p* < 0.001), and incomplete vaccination (26.5% vs. 16.9%, *p* < 0.001). The condition was more common in individuals with prior metabolic disorders (38.0% versus 23.3%), and obesity (47.0% compared with 29.4%). The most common long-lasting symptoms included fatigue (68% of cases), dyspnea (45%), and cognitive impairment (39%), while 41% required specialist care and 12% work disability.

The strong predictor, with moderate and severe COVID-19 cases showing 3 times the odds of long COVID (OR = 2.92, 95% CI: 2.38–3.58, *p* < 0.001). In addition, incomplete vaccination status increased the risk (OR = 1.77, 95% CI: 1.40–2.24, *p* < 0.001). Among comorbidities, metabolic disorders (OR = 2.02, 95% CI: 1.63–2.50), obesity (OR = 2.13, 95% CI: 1.74–2.61), and all demonstrated significant associations with Long COVID development (all *p* < 0.001).

The symptom cluster analysis revealed particularly striking findings. Participants with persistent cardiopulmonary symptoms (dyspnea) were 10.3 times more likely (95% CI: 7.89–13.5; *p* < 0.001), while those with neurological manifestations (cognitive impairment) were 11.8 times more likely (11.8 (95% CI: 8.73–15.9)). meet the long COVID criteria compared to those whose symptoms have resolved within three months (Table 2).

Table 3 show the summary of Independent Predictors of Long COVID After adjusting, moderate to severe acute infection (aOR 2.5, 95% CI 2.0–3.1, *p* < 0.001) and female sex (aOR 1.8, 95% CI 1.4–2.3, *p* < 0.01) remained significant contributors.

Female sex remained independently associated with Long COVID (aOR 1.8, 95% CI 1.4–2.3, *p* < 0.01), moderate-to-severe acute infection (aOR = 2.5, 95% CI: 2.0–3.1, *p* < 0.01) and pre-existing metabolic disorders (aOR = 1.6, 95% CI: 1.2–2.1, *p* < 0.05). Other independent risk factors included rural residence (aOR 1.3, 95% CI 1.0–1.7, *p* = 0.04), incomplete vaccination (aOR 1.6, 95% CI 1.2–2.1, *p* < 0.01), and obesity (aOR 1.5, 95% CI 1.2–1.9, *p* < 0.01). Each 10-year increase in age was associated with a 20% increase in risk (aOR = 1.2, *p* = 0.003).

Table 4 presents subgroup analyses. Females had higher rates of severe fatigue than males (73% vs. 59%, *p* < 0.001). Metabolic comorbidities exacerbated symptom severity (75% fatigue with disorders, 64% without) and doubled specialist care needs (53% vs. 34%, *p* < 0.001). Worse outcomes. were reported among the unvaccinated and rural residents (all *p* < 0.05).

**Figure 2:**
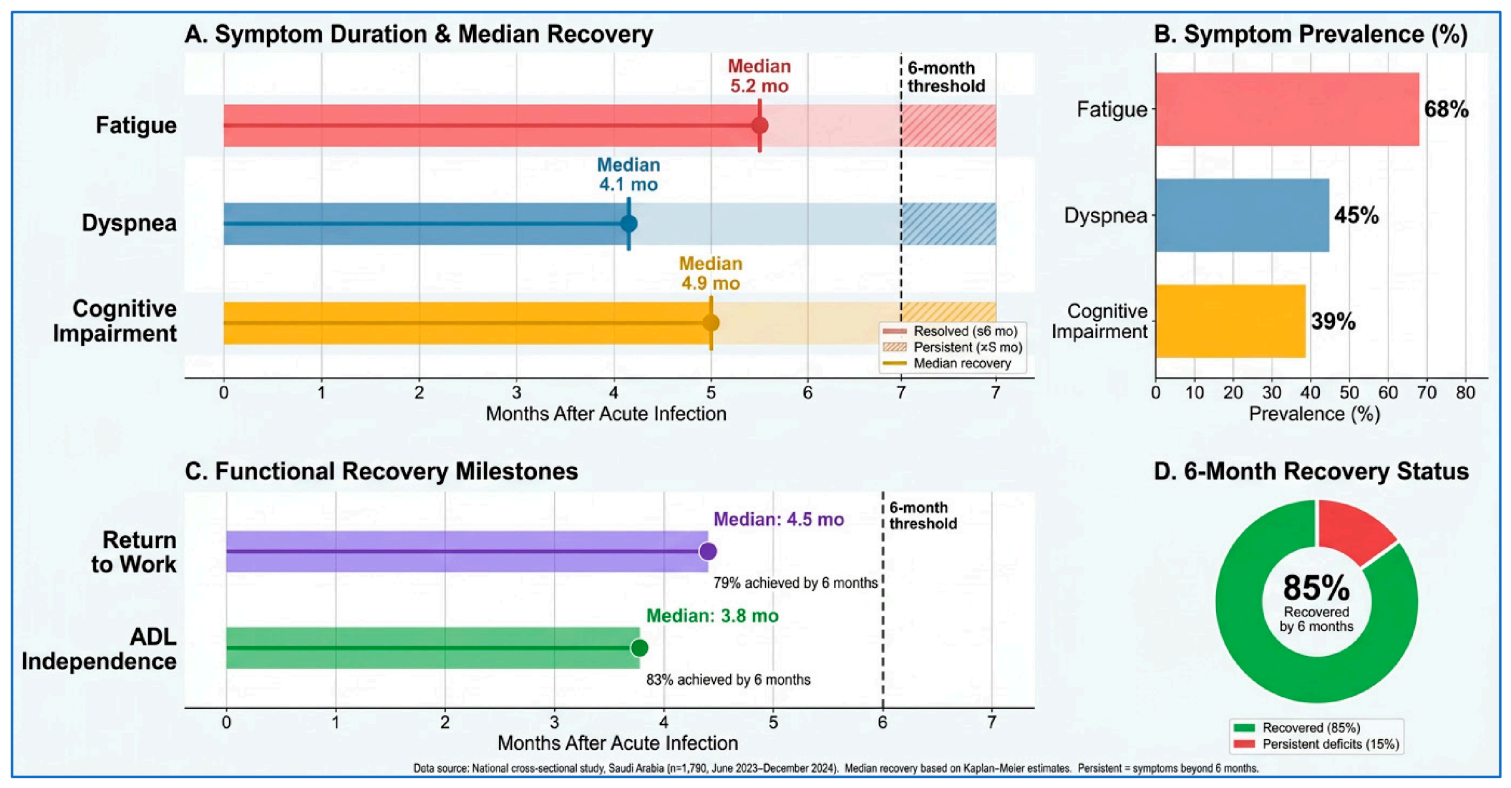
Longitudinal trajectories of symptom persistence and functional recovery among Long COVID participants over 6 months (retrospective recall, *n* = 573). (**A**) Median time to recovery for fatigue, dyspnea, and cognitive impairment. (**B**) Symptom prevalence at baseline, 3 months, and 6 months. (**C**) Functional recovery (ADL independence and return to work). (**D**) Overall recovery landscape showing bimodal outcomes.

Data source for symptom trajectories: The symptom persistence and recovery data presented in Table 5 and Figure 2 are based on participants’ retrospective recall during the cross-sectional survey. Participants were asked to remember whether each symptom was present at 3 months, 6 months, and 9 months after their acute infection, and if resolved, the approximate month of resolution. Therefore, these data represent recalled estimates, not prospective measurements.

**Figure 3:**
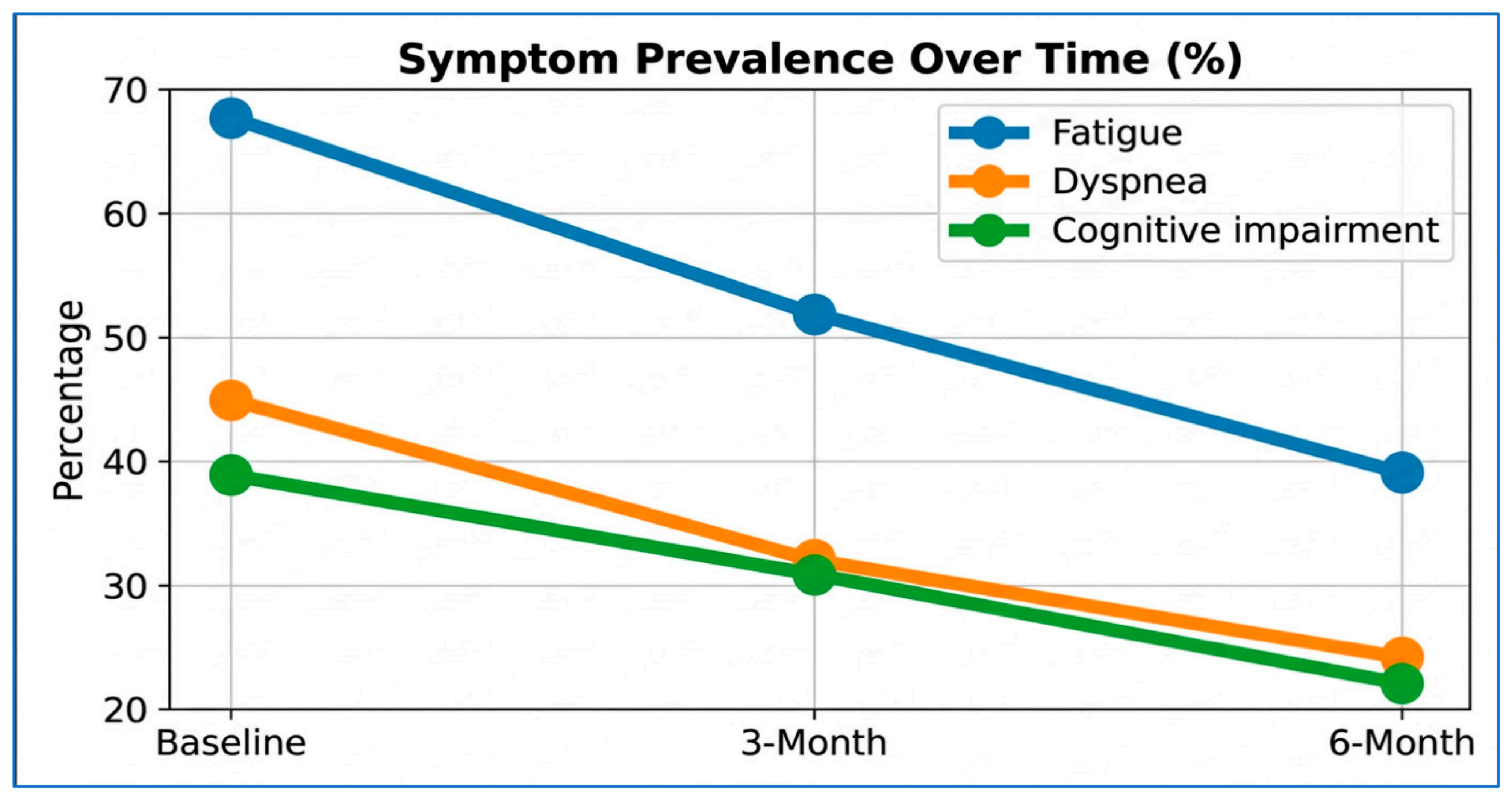
Symptoms over time.

Table 5 and Figure 3 show symptom trajectories over 6 months. Fatigue prevalence declined from 68% at baseline to 39% at 6 months (*p* < 0.001). Regarding cognitive dysfunction, 78% resolved within 6 months, while 15% of patients had persistent deficits, possibly reflecting distinct neurological phenotypes.

### 3.1. Multivariable Predictors of Long COVID Development

**Figure 4:**
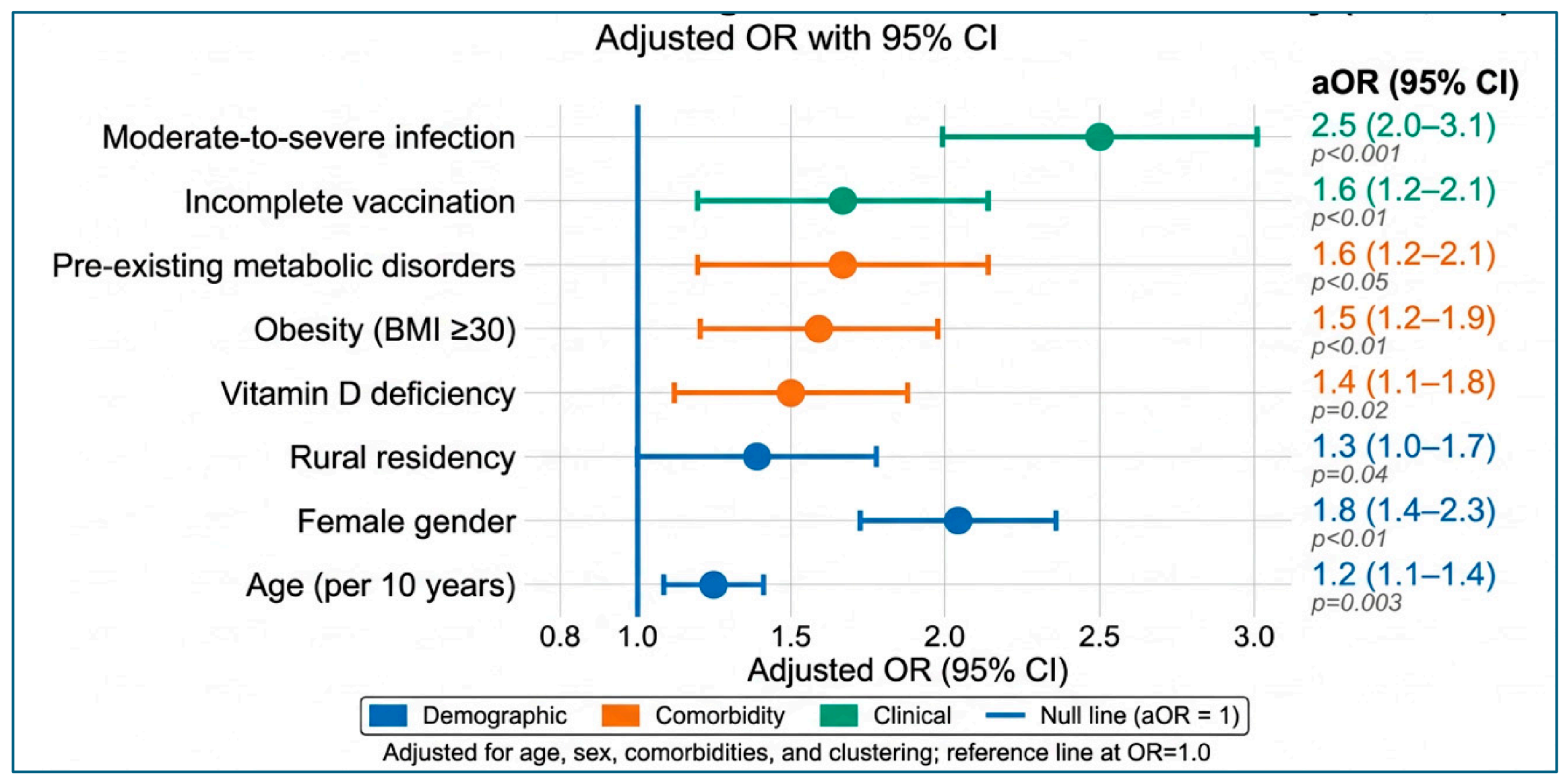
Multivariate risk factors for long covid, Saudi Arabia (*n* = 1790).

The final multivariable logistic regression analysis explaining independent predictors of Long COVID among 1790 PCR-confirmed cases is displayed in Figure 4. Forest plot of 8 significant risk factors (adjusted odds ratios [aOR] and 95% confidence intervals). The model demonstrates reasonable discrimination (AUC = 0.78), calibration (Hosmer-Lemeshow *p* = 0.42), and explains 28% of the variance (Nagelkerke R^2^ = 0.28). Each confidence interval is entirely above 1.0, confirming that each predictor variable makes an independent contribution.

The strongest risk factor identified was moderate-to-severe acute infection (aOR = 2.5, 95% CI 2.0–3.1, *p* < 0.001). Female sex (aOR = 1.8, 95% confidence interval (CI): 1.4; 2.3; *p* < 0.01), incomplete vaccination status (aOR = 1.6, CI 95%: 1.2; 2; *p* < 0.01) and preexisting metabolic diseases (aOR = 1.6, CI 95%: 1.2–2.1; *p* < 0.05), were associated with outcomes to similar extents Obesity (BMI ≥ 30) also remained significant (aOR = 1.5, 95% CI 1.2–1.9, *p* < 0.01). Rural residency (vs urban) was associated with a 30% increased odds (aOR = 1.3, 95% CI, 1.0–1.7, *p* = 0.04), and each decade of age increment was associated with an increased risk of 20% (aOR = 1.2, 95% CI: 0.1–1.4; *p* = 0.003).

### 3.2. Temporal Dynamics of Symptom Persistence and Functional Recovery

Tabulated summary of symptom progression and recovery patterns among the 573 Long COVID participants is shown in Figure 4 (four-panel plot). Median recovery times from fatigue (5.2 months, 68%), dyspnea (4.1 months, 45%), and cognitive impairment (4.9 months, 39%) are shown in Panel A. Notably, ~15% of participants had long-term deficits, underscoring a chronic subgroup. Panel B shows that fatigue is the most common symptom, followed by dyspnea and cognitive impairment.

The 39% with moderate-to-severe symptoms at six months are shown in Panel C, but the majority became functionally independent (83% ADL independent; median time to independence 3.8 months) and returned to work (79%; median return date 4.5 months). Panel D shows the bimodal recovery pattern: 85% recovered or improved at six months, while 15% suffered severe disability.

This bimodal course has important clinical implications. One possible solution is to provide chronic care models specifically for the 15% of Americans who suffer from persistent deficits. Second, functional recovery (i.e., ADL independence and RTW) usually precedes full symptomatic recovery in most cases; thus, rehabilitation for functional restoration, plus consolidation at work, is a priority. What these results mean to you: These findings can be useful when educating patients, making clinical decisions, and developing longitudinal follow-up protocols.

### 3.3. Geographic Variation in Long COVID Prevalence Across Saudi Arabia

**Figure 5:**
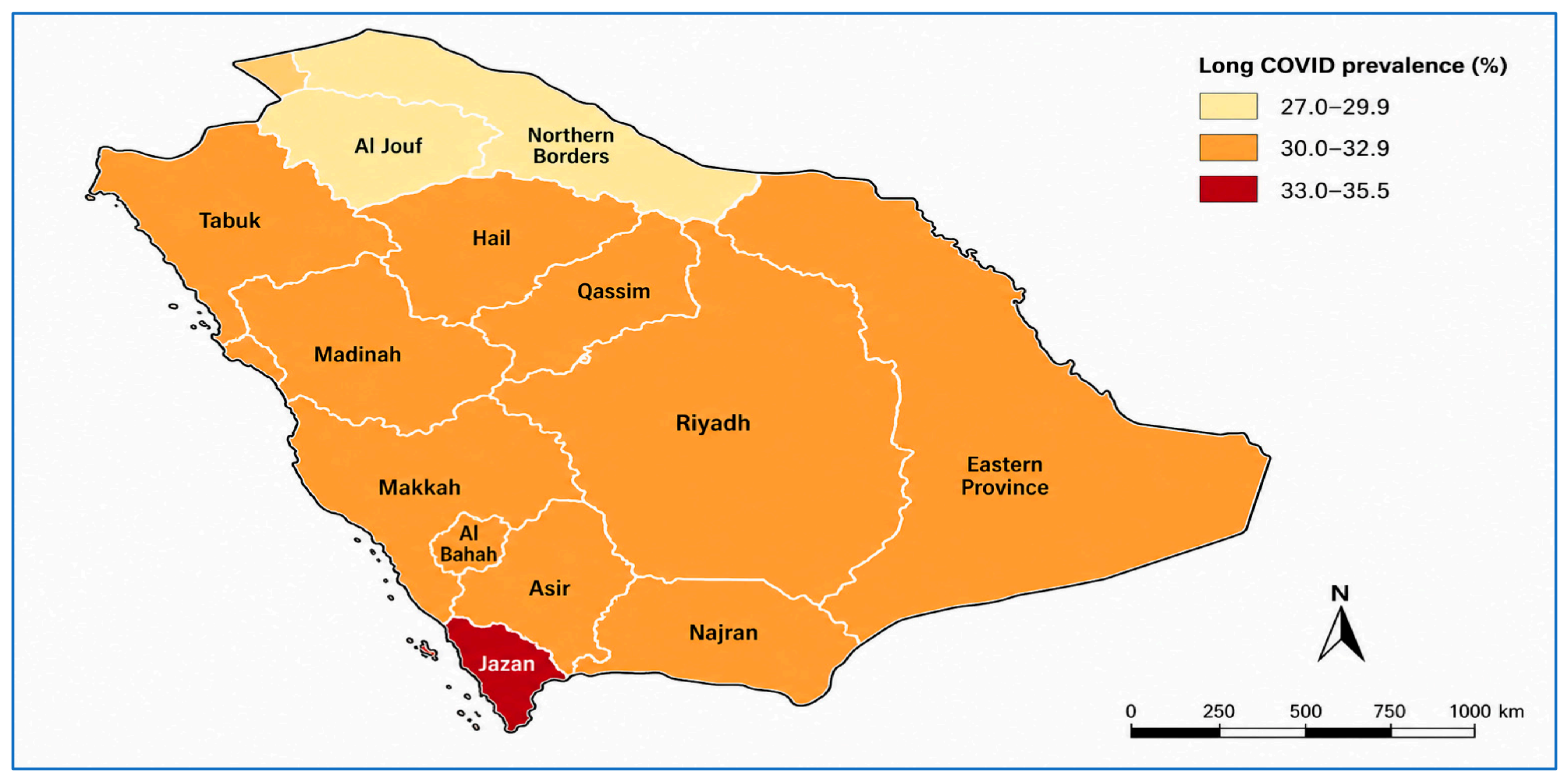
Geographic distribution of Long COVID prevalence across the 13 administrative regions of Saudi Arabia (*n* = 1790).

The geographic distribution of Long COVID prevalence throughout the 13 administrative regions of Saudi Arabia is depicted in Figure 5. The national prevalence varied from 27.0% at Al-Jouf to 35.5% in Jazan, an absolute inter-regional difference of 8.5 percentage points. Prevalence estimates were close to the national estimate of 32.0% across most regions. At the same time, the burden was highest in Jazan and lowest in Al-Jouf and Northern Borders—administrative boundaries taken from official GIS data obtained by the General Authority for Statistics.

Qualitative analysis (Table 6) showed the qualitative findings. Four themes were identified: (1) debilitating and debilitating/debilitating symptomatology; (2) systemic health barriers, such as diagnostic delays and disparities in rural areas; (3) significant social impacts; and 4 patient-generated coping strategies, such as pacing. Participants described cognitive dysfunction and burnout, while their stories of workplace stigma explained the 12% disability rate. Rural participants emphasized access, access restrictions more than expected, given the lingering risk of Covid. These findings highlight the need to (1) educate clinicians to avoid delays in diagnosis, (2) expand rehabilitation services in rural areas, and (3) protect vulnerable patients in the workplace.

## 4. Discussion

This national cross-sectional study represents the first large-scale investigation of Long COVID epidemiology in Saudi Arabia, encompassing 1790 adults with PCR-confirmed COVID-19 infection across all 13 administrative regions.

### 4.1. Long COVID Impact Prevalence in the Context

The prevalence of 32.0% in Saudi Arabia is in the middle range of global estimates, which vary widely from 20% to 54%, reflecting differences in case definitions, follow-up duration, and population characteristics [6,17]. A global epidemiology study review by Song et al. From 51 countries, pooled median prevalence estimates ranged from 43% at 3 months post-infection, with considerable heterogeneity explained in part by differences in the methodologies applied and the capacities of healthcare systems [18]. Our results are consistent with these demographic and metabolic profiles across the Saudi population compared with international benchmarks [19].

According to the literature, about a third of the Kingdom’s COVID-19 survivors experience similar long-term consequences. Our study partially addressed some of these shortcomings by using WHO-standardized definitions, dual-mode data collection to increase representativeness, and a comprehensive risk-factor assessment, thereby providing an important benchmark for future Saudi and regional studies [20].

### 4.2. Symptom Profile: Fatigue, Dyspnea, and Cognitive Impairment

The triad of fatigue (68%), dyspnea (45%), and cognitive impairment (39%) observed in our series highlights the patterns of symptom occurrence, underscoring the multisystem nature of LTV. Fatigue was the most frequent, with an average duration of 5.2 months, the longest of all symptoms. A recent study showed that persistent fatigue affected 20–30% of symptomatic COVID-19 survivors at six months, with higher rates in women and those with severe acute illness [21].

Dyspnea, which affects 45% of our cohort, reflects the cardiopulmonary consequences of COVID-19 infection, while dyspnea resolved faster than fatigue (4.1 months on average), the fact that almost a quarter of patients persisted for six months highlights the importance of ongoing monitoring. This may encourage doctors to treat lung or cardiovascular disease in the long, long term. The high prevalence of dyspnea in our population—compared to the 40–50% rate seen in hospital patient populations worldwide—points to the need for pulmonary rehabilitation programs and longitudinal spirometry monitoring [22].

These findings show that Long COVID effects are heterogeneous, with different clinical phenotypes emerging across demographic and clinical strata.

### 4.3. Determinants of Long COVID

Our findings demonstrate notable demographic and clinical differences in susceptibility to Long COVID among the Saudi population, underscoring potential targets for prevention and management.

The model showed good discrimination (AUC = 0.78) and calibration (Hosmer-Lemeshow *p* = 0.42), accounting for ~28% of the variation in development of Long COVID (Nagelkerke R^2^ = 0.28). These results validate that demographic, clinical, and metabolic factors act independently of their univariate associations in Long COVID susceptibility.

#### 4.3.1. Female Sex

Significant demographic differences with female participants having 1.82-fold greater odds of Long COVID than males (95% CI: 1.48–2.24, *p* < 0.001).

Female sex was the only consistently strong independent predictor of Long COVID (aOR = 1.8, 95% CI 1.4–2.3), in keeping with global meta-analyses and systematic reviews [21]. Tsampasian et al. synthesized data from 41 studies and found a pooled odds ratio for female sex of 1.56 (95% CI 1.41–1.73), explaining this difference by hormonal, immunological, and psychosocial factors [23,24].

The synthesis of international and local data points to an urgent need for sex-disaggregated care pathways, including tailored rehabilitation protocols based on the biological aspects of cardiac allograft rejection/acceptance, as well as hormonal evaluations and psychosocial support tailored to mitigate a clear gendered burden that we have identified in this cohort.

#### 4.3.2. Acute Disease Severity

Initially, the strongest correlate of Long COVID in our multivariable model was moderate-to-severe acute infection (aOR = 2.5, 95% CI 2.0–3.1), supporting findings from the global literature. Patients with severe acute illness are thought to have higher viral load, more vigorous cytokine storms, and resultant end-organ injury leading to susceptibility to prolonged sequelae [2].

Global prevalence studies highlighted the severity and PASC. Wulf Hanson et al. reported higher odds of Long COVID among hospitalized patients (2–3 times higher than in non-hospitalized individuals) and that intensive care unit admission conferred the highest risk [22]. Specifically in the Saudi setting, Alkwai et al. noted that when moderate-to-severe acute infection was compared, delayed return to usual health occurred with a median recovery time of more than 6 months [25]. Alradini et al. also found that hospitalization for acute COVID-19 doubled the risk of post-acute manifestations in a nationally representative Saudi cohort [17]. Our findings support the crucial role of acute-phase interventions, such as early antiviral therapy, immunomodulation, and supportive care, in preventing long-term sequelae.

#### 4.3.3. Metabolic Disorders and Obesity

Some factors were independent predictors, including pre-existing metabolic disorders (aOR = 1.6, 95% CI 1.2–2.1) and obesity (aOR = 1.5, 95% CI 1.2–1.9), in keeping with the very high prevalence of those conditions in Saudi Arabia (28% and 35% of our sample, respectively).

Chronic low-grade inflammation and associated insulin resistance and endothelial dysfunction in metabolic disorders, including diabetes mellitus, hypertension, and dyslipidemia, could exacerbate COVID-19 induced tissue injury, limiting recovery [21].

In our own series, patients with metabolic disorders had a significantly worse outcome: 75% reported severe fatigue (compared with 64% without such disorders), 53% required a specialist visit (compared with 34%), and 18% were unable to work, while only 8% did not suffer from such disorders. These gaps highlight how long-term coronavirus-related metabolic diseases exacerbate the severity and health burden. Saudi-specific evidence supports these findings.

#### 4.3.4. Incomplete Vaccination

The risk of Long COVID was significantly associated with incomplete vaccination status (unvaccinated or partially vaccinated) (1.6 [95% CI 1.2–2.1]), indicating a protective effect of complete vaccination. Vaccines decrease the risk of Long COVID via several mechanisms: they decrease acute viral burden, dampen inflammatory processes, reduce disease severity, and may prevent viral persistence. Tsampasian et al. synthesized evidence from 12 studies and found that full vaccination reduced the risk of Long COVID by about 40–50%, with booster doses providing further protection [16]. Our findings further emphasize the need for booster vaccination campaigns targeting high-risk subgroups (females, obese, metabolically compromised) and underserved groups (rural residents) to reduce Long COVID incidence and severity. Public health should focus on vaccine protection against not only acute illness but also debilitating sequelae, thereby increasing vaccine uptake and adherence.

#### 4.3.5. Rural Residency

Geographic and socioeconomic factors were significant, with rural living. Rural residency was an independent predictor of Long COVID (aOR = 1.3; 95% CI 1.0, 1.7)—a novel finding that has important implications for equity. In Saudi Arabia, barriers to healthcare access for Long COVID patients are multifactorial, including limited availability of hospitals with specialists, longer distances patients must travel, delays in diagnostics, and a lack of awareness.

Little attention has been given to the rural-urban disparity in Long COVID outcomes in the global literature. Kim et al. established sociodemographic predictors of Long COVID in the United States, such as lower socioeconomic status and diminished healthcare access, which mirror rural disadvantage [20]. Especially in the rural areas, low physician density and insufficient rehabilitation facilities and telehealth infrastructure may be delaying both diagnosis as well as the treatment process, which can lead to prolonged symptoms resulting in deterioration of outcome [2]. Alenezi et al. characterized the development of Saudi virtual post-COVID clinics as a scheme to cross rural-urban divides through remote visits, symptom tracking, and rehabilitative counseling [26]. Our findings underscore the need to expand telehealth services, support training for rural primary care providers in low-resource areas to recognize and manage Long COVID and establish mobile or satellite specialty clinics. Rural disparity is not just about achieving patient outcomes but about reaching our Vision 2030 national health equity goal—a goal the country must continue to strive for.

### 4.4. Healthcare and Socioeconomic Burden

Long COVID placed significant health care and socioeconomic costs on our cohort, with 41% of those affected requiring specialty care and 12% reporting work disability. These numbers are consistent with global estimates: Lau et al. the proportion of Long COVID in a US cohort that required multiple specialist visits was reported to be 30–40%, while disability due to physical and mental fatigue affected 15–20% [27]. Recognizing such systemic challenges can encourage policymakers to prioritize infrastructure development, workforce expansion, and policy reforms to support long-term COVID-19 recovery efforts.

Based on our findings, we recommend a comprehensive multi-stakeholder framework to mitigate Long COVID throughout Saudi Arabia. This framework comprises (1) Ministry of Health funding and coordination of multidisciplinary clinics; (2) well-trained primary care providers for early recognition using WHO case definitions; (3) specialty services to provide integrated rehabilitation (pulmonary, neurological and cardiac); (4) workplace accommodation policies to reduce the 12% work disability burden by flexible hours, phased return to work; and (5) patient self-management support including pacing strategies with peer support networks. We believe an integrated approach is important, given the 41% share of specialty care demand in our cohort.

## 5. Limitations

Several limitations should be acknowledged. Due to the cross-sectional design, we can only ascertain associations; reverse causation and residual confounding are possible. Self-reported information about symptoms and comorbidities is subject to recall bias. Over-representation of younger people and under-representation of more digitally deprived people may have introduced selection bias through concurrent use of online and telephone recruitment. Without objective clinical assessment (e.g., pulmonary function tests, biomarkers), there is a risk of misclassification. The exclusion of dead or severely ill patients may also create a bias in the findings. The absence of a control group without COVID-19 impedes attributing all lasting symptoms to COVID-19 alone. In addition, 6-month symptom trajectories were based on retrospective recall instead of prospective follow-up. As a result, the findings should be understood as hypothesis-generating and need to be verified in prospective cohort studies.

## Figures and Tables

**Table 1:** Baseline Characteristics and Long COVID Prevalence (*n* = 1790).

Characteristic	Total Sample (*n* = 1790)	Long COVID Cases (*n* = 573, 32.0%)	Non-Long COVID (*n* = 1217, 68.0%)	*p*-Value
Demographics				
Age (mean ± SD)	45.2 ± 12.8	47.5 ± 11.6	44.1 ± 13.2	<0.001
Gender				
Male	859 (48.0%)	218 (38.0%)	641 (52.7%)	<0.001
Female	931 (52.0%)	355 (62.0%)	576 (47.3%)	
Ethnicity/Nationality				
Saudi	1253 (70.0%)	421 (73.5%)	832 (68.4%)	0.03
Non-Saudi	537 (30.0%)	152 (26.5%)	385 (31.6%)	
Residency				
Urban	1433 (80.1%)	442 (77.1%)	991 (81.4%)	0.02
Rural	357 (19.9%)	131 (22.9%)	226 (18.6%)	
Occupation				
Employed	1201 (67.1%)	342 (59.7%)	859 (70.6%)	<0.001
Unemployed	589 (32.9%)	231 (40.3%)	358 (29.4%)	
Clinical Features				
Acute COVID-19 Severity				
Mild	1164 (65.0%)	275 (48.0%)	889 (73.0%)	<0.001
Moderate-to-severe	626 (35.0%)	298 (52.0%)	328 (27.0%)	
Vaccination Status				
Fully vaccinated	1432 (80.0%)	421 (73.5%)	1011 (83.1%)	<0.001
Unvaccinated/Partial	358 (20.0%)	152 (26.5%)	206 (16.9%)	
Comorbidities				
Metabolic disorders	501 (28.0%)	218 (38.0%)	283 (23.3%)	<0.001
Obesity (BMI ≥ 30)	627 (35.0%)	269 (47.0%)	358 (29.4%)	<0.001
Cardiovascular disease	269 (15.0%)	120 (21.0%)	149 (12.2%)	<0.001
Long COVID Symptoms *	—		—	—
Fatigue	—	390 (68.0%)	89 (7.3%)	—
Dyspnea	—	258 (45.0%)	62 (5.1%)	—
Cognitive impairment	—	223 (39.0%)	47 (3.9%)	—
Healthcare Burden	—		—	—
Required specialty care	—	235 (41.0%)	—	—
Work disability	—	69 (12.0%)	—	—

* Symptom present during acute infection but resolved by 3 months almost three times increased odds for developing Long COVID (OR = 2.92, 95% CI: 2.38–3.58, *p* < 0.001). Incompleteness of vaccination also conferred a greater risk (OR = 1.77, 95% CI: 1.40–2.24, *p* < 0.001).

**Table 2:** Risk Factors of Long COVID (Unadjusted Analysis).

Risk Factor	Long COVID (*n* = 573)	Non-Long COVID (*n* = 1217)	Unadjusted OR (95% CI)	*p*-Value
Demographic Factors				
Female sex	355 (62.0%)	576 (47.3%)	1.82 (1.48–2.24)	<0.001
Rural residency	131 (22.9%)	226 (18.6%)	1.31 (1.03–1.67)	0.03
Unemployed status	231 (40.3%)	358 (29.4%)	1.63 (1.33–2.00)	<0.001
Clinical Factors				
Moderate-to-severe infection	298 (52.0%)	328 (27.0%)	2.92 (2.38–3.58)	<0.001
Unvaccinated/Partial	152 (26.5%)	206 (16.9%)	1.77 (1.40–2.24)	<0.001
Comorbidities				
Metabolic disorders	218 (38.0%)	283 (23.3%)	2.02 (1.63–2.50)	<0.001
Obesity (BMI ≥ 30)	269 (47.0%)	358 (29.4%)	2.13 (1.74–2.61)	<0.001
Symptom Clusters				
Cardiopulmonary (Dyspnea)	258 (45.0%)	89 (7.3%)	10.3 (7.89–13.5)	<0.001
Neurological (Cognitive)	223 (39.0%)	62 (5.1%)	11.8 (8.73–15.9)	<0.001

Reference group: Non-Long COVID participants reporting the symptom during acute infection but resolving by 3 months.

**Table 3:** Independent Risk Factors for Long COVID: Multivariable Logistic Regression Analysis.

Variable	Adjusted OR	95% CI	*p*-Value	Reference Category
Demographic Factors				
Age (per 10-year increase)	1.2	1.1–1.4	0.003	Continuous
Female gender	1.8	1.4–2.3	<0.01	Male
Rural residency	1.3	1.0–1.7	0.04	Urban
Clinical Factors				
Moderate-to-severe acute infection	2.5	2.0–3.1	<0.01	Mild infection
Unvaccinated/partially vaccinated	1.6	1.2–2.1	<0.01	Fully vaccinated
Comorbidities				
Pre-existing metabolic disorders	1.6	1.2–2.1	<0.05	No metabolic disorders
Obesity (BMI ≥ 30)	1.5	1.2–1.9	<0.01	BMI < 30

**Table 4:** Subgroup Analysis of Long COVID Symptom Severity, Functional Impairment, and Healthcare Utilization.

Subgroup	Prevalence of Severe Symptoms (%)	Functional Impairment (%)	Healthcare Utilization (%)	Work Disability (%)	*p*-Value (vs. Reference)
	(Fatigue/Dyspnea/Cognitive)	(ADL Limitations)	(≥3 Specialist Visits)	(Unable to Work)	
Overall Long COVID	68/45/39	32	41	12	—
By Gender					
Female (*n* = 355)	73/49/44	38	47	15	<0.01
Male (*n* = 218)	59/38/30	22	31	7	(ref)
By Acute Severity					
Moderate-Severe (*n* = 298)	78/58/51	43	55	19	<0.001
Mild (*n* = 275)	57/31/25	20	26	4	(ref)
By Metabolic Status					
With Disorder (*n* = 218)	75/54/47	40	53	18	<0.001
Without (*n* = 355)	64/40/34	27	34	8	(ref)
By Vaccination					
Unvaccinated (*n* = 152)	72/53/46	39	50	17	<0.01
Fully Vaccinated (*n* = 421)	66/42/36	29	38	10	(ref)
By Residency					
Rural (*n* = 131)	71/50/43	37	48	16	0.02
Urban (*n* = 442)	67/43/37	30	39	11	(ref)

Key: ADL Limitations: Activities of Daily Living requiring assistance. Prevalence of Severe Symptoms: Percentage with frequent/severe symptoms (≥4 days/week). Reference groups: Male, mild infection, no metabolic disorders, fully vaccinated, urban residency. Statistical tests: Chi-square for proportions, ANOVA for continuous variables.

**Table 5:** Longitudinal Trends in Long COVID Symptom Persistence and Recovery.

Variable	Baseline (*n* = 573)	3-Month (*n* = 522)	6-Month (*n* = 487)	*p*-Value (Trend Test)	Median Time to Recovery (95% CI)
Symptom Prevalence					
Fatigue	390 (68%)	271 (52%)	190 (39%)	<0.001	5.2 mo (4.8–5.6)
Dyspnea	258 (45%)	167 (32%)	117 (24%)	<0.001	4.1 mo (3.7–4.5)
Cognitive impairment	223 (39%)	162 (31%)	107 (22%)	<0.001	4.9 mo (4.3–5.5)
Symptom Severity					
Mild	160 (28%)	188 (36%)	219 (45%)	<0.001	—
Moderate	298 (52%)	250 (48%)	190 (39%)	0.003	—
Severe	115 (20%)	84 (16%)	54 (11%)	0.01	—
Functional Recovery					
Full ADL independence	390 (68%)	397 (76%)	404 (83%)	<0.001	3.8 mo (3.4–4.2)
Return to work (if employed)	194/342 (54%)	232/342 (68%)	270/342 (79%)	<0.001	4.5 mo (4.1–4.9)
Healthcare Utilization					
≥1 Specialist visit/month	235 (41%)	167 (32%)	112 (23%)		

**Table 6:** Qualitative Themes from Lived Experiences of Long COVID (*n* = 420 Open-Ended Responses).

Theme	Subtheme	Representative Quotes	Link to Quantitative Findings	Participant Characteristics
Symptom Burden	Multisystem Fluctuations	“Some days I can walk; other days, I can’t get out of bed due to fatigue and leg pain.”	Aligns with high fatigue (68%) and disability (12%)	Female, 42, urban, moderate acute COVID
	Cognitive Dysfunction	“I forget words mid-sentence—my brain feels like it’s full of fog.”	Supports 39% cognitive impairment prevalence	Male, 35, rural, severe acute COVID
Healthcare Access	Diagnostic Challenges	“Doctors said it’s anxiety, but my chest pain is real.”	Explains high specialist care needs (41%)	Female, 50, urban, metabolic disorder
	Rural Disparities	“No clinics nearby understand Long COVID. I travel 3 h for treatment.”	Contextualizes rural residency risk (aOR = 1.3)	Male, 28, rural, unvaccinated
Social Impact	Workplace Stigma	“My employer thinks I’m lying. I lost my job after 3 months of sick leave.”	Correlates with 12% work disability	Female, 38, urban, employed at baseline
	Family Strain	“My kids don’t understand why I can’t play with them anymore.”	Highlights ADL limitations (32%)	Male, 45, rural, obese
Coping Strategies	Self-Management	“I pace my activities—doing 10% of what I used to—to avoid crashes.”	Reflects moderate-severe symptom persistence (39%)	Female, 30, urban, vaccinated
	Peer Support	“Online groups saved me; they’re the only ones who believe my symptoms.”		

## Data Availability

The data used in this study are available from the corresponding author upon request.

## Supplementary Materials

The following supporting information can be downloaded at: https://www.mdpi.com/article/10.3390/jcm15155902/s1, File S1: STROBE Statement—checklist of items that should be included in reports of observational studies.

## Abbreviations

ADL	Activities of Daily Living.
aOR	Adjusted Odds Ratio.
AUC	Area Under the Curve.
BMI	Body Mass Index.
CI	Confidence Interval.
CME	Continuing Medical Education.
COVID-19	Coronavirus Disease 2019.
GP	General Practitioner.
IQR	Interquartile Range.
IRB	Institutional Review Board.
MENA	Middle East and North Africa.
OR	Odds Ratio.
PASC	Post-Acute Sequelae of SARS-CoV-2 Infection.
PCR	Polymerase Chain Reaction.
REDCap	Research Electronic Data Capture.
RT-PCR	Reverse Transcription Polymerase Chain Reaction.
SD	Standard Deviation.
WHO	World Health Organization.

## References

[1] Hou Y., Gu T., Ni Z., Shi X., Ranney M.L., Mukherjee B. (2025). Global Prevalence of Long COVID, its Subtypes, and Risk factors: An Updated Systematic Review and Meta-analysis. Open Forum Infect. Dis..

[2] Alenezi F., Alsagheir A., Amer S., Alzubaidi L., Alhomod A.A.S., Alamaa T. (2025). Virtual post-COVID-19 clinics in Saudi Arabia: Navigating the effect of the pandemic with a national project. Front. Public Health.

[3] Luo D., Mei B., Wang P., Li X., Chen X., Wei G., Kuang F., Li B., Su S. (2023). Prevalence and risk factors for persistent symptoms after COVID-19: A systematic review and meta-analysis. Clin. Microbiol. Infect..

[4] Halas R.-G., Vaduva D.M.B., Radulescu M., Bredicean A.-C., Mateescu D.-M., Toma A.-O., Cotet I.-G., Guse C.-E., Marginean A., Margan M.-M. (2025). Long COVID Prevalence and Risk Factors: A Systematic Review and Meta-Analysis of Prospective Cohort Studies. Biomedicines.

[5] Alkwai H.M., Khalifa A.M., Ahmed A.M., Alnajib A.M., Alshammari K.A., Alrashidi M.M., Ahmed H.G. (2022). Persistence of COVID-19 symptoms beyond 3 months and the delayed return to the usual state of health in Saudi Arabia: A cross-sectional study. SAGE Open Med..

[6] Samannodi M., Alwafi H., Naser A.Y., Al Qurashi A.A., Qedair J.T., Salawati E., Almatrafi M.A., Ekram R., Bukhari R.I., Dahlawi M. (2022). Determinants of Post-COVID-19 Conditions among SARS-CoV-2-Infected Patients in Saudi Arabia: A Web-Based Cross-Sectional Study. Diseases.

[7] Kamal S.M., Al Qahtani M.S., Al Aseeri A., Naghib M.E., Al Mazroua A.M., Alshamrani A.M., Al Mazroua M.M., AlHarbi F.S. (2025). Long COVID-19: A Four-Year prospective cohort study of risk factors, recovery, and quality of life. BMC Infect. Dis..

[8] Han Q., Zheng B., Daines L., Sheikh A. (2022). Long-Term Sequelae of COVID-19: A Systematic Review and Meta-Analysis of One-Year Follow-Up Studies on Post-COVID Symptoms. Pathogens.

[9] Dale Z., Wallington S.F., Penn-Marshall M. (2025). Prevalence and characteristics of post-acute sequelae of COVID-19 in recovered patients. Front. Public Health.

[10] Alharbi A., Almogbel F., Rabbani U., Memish Z.A. (2024). Long COVID-19 and coexistence of fatigue and depression: A Cross-sectional study from Saudi Arabia. J. Epidemiol. Glob. Health.

[11] Nittas V., Gao M., West E.A., Ballouz T., Menges D., Hanson S.W., Puhan M.A. (2022). Long COVID through a public health lens: An umbrella review. Public Health Rev..

[12] Yuan N., Lv Z.H., Sun C.R., Wen Y.Y., Tao T.Y., Qian D., Tao F.P., Yu J.H. (2023). Post-acute COVID-19 symptom risk in hospitalized and non-hospitalized COVID-19 survivors: A systematic review and meta-analysis. Front. Public Health.

[13] Aldhawyan A.F., BuSaad M.A., Almaghlouth N.E., Alnasser A.H., Alnasser J.A., Almansour A.H., AlHarkan K.S. (2024). Under-standing long COVID: Prevalence, characteristics, and risk factors in the Eastern Province of Saudi Arabia. Front. Med..

[14] Al-Johani M.S., Khalil R., Al-Mohaimeed Y.A., Al-Mundarij O.M., Al-Samani A.S., Al-Saqry O.S., Al-Saawi A.A., Al-Dhali I.K., Al-Essa W.A. (2023). Post-COVID-19 fatigue and health-related quality of life in Saudi Arabia: A population-based study. Front. Public Health.

[15] Almalag H.M., Altuwaijri N., Alnaim L.S., Alassiri D., Alsolaimi G., Aldakhil S., Al Aloola N., Alsabhan J.F., Bawazeer G.A., Al Juffali L. (2025). Prevalence and characteristics of long COVID among COVID-19 survivors in Saudi Arabia: A cross-sectional study. IJID Reg..

[16] Wynberg E., Han A.X., van Willigen H.D., Verveen A., van Pul L., Maurer I., van Leeuwen E.M., van den Aardweg J.G., de Jong M.D., Nieuwkerk P. (2023). Inflammatory profiles are associated with long COVID up to 6 months after illness onset: A prospective cohort study of individuals with mild to critical COVID-19. medRxiv.

[17] Alradini F.A., Alamri F., Aljahany M.S., Almuzaini Y., Alsofayan Y., Khan A., Albogami N., Abdulrahim M., Almogbil A., Alahmari A. (2022). Post-acute COVID-19 condition in Saudi Arabia: A national representative study. J. Infect. Public Health.

[18] Song X., Song W., Cui L., Duong T.Q., Pandey R., Liu H., Zhou Q., Sun J., Liu Y., Li T. (2024). A Comprehensive Review of the Global Epidemiology, Clinical Management, Socio-Economic Impacts, and National Responses to Long COVID with Future Research Directions. Diagnostics.

[19] Maglietta G., Diodati F., Puntoni M., Lazzarelli S., Marcomini B., Patrizi L., Caminiti C. (2022). Prognostic Factors for Post-COVID-19 Syndrome: A Systematic Review and Meta-Analysis. J. Clin. Med..

[20] Wahyudi A. (2024). The Impact of Long COVID on Global Public Health: Long-term Consequences and Healthcare System Preparedness. Magenta J. Heal..

[21] Wulf Hanson S., Abbafati C., Aerts J.G., Al-Aly Z., Ashbaugh C., Ballouz T., Blyuss O., Bobkova P., Bonsel G., Global Burden of Disease Long COVID Collaborators (2023). Estimated Global Proportions of Individuals with Persistent Fatigue, Cognitive, and Respiratory Symptom Clusters Following Symptomatic COVID-19 in 2020 and 2021. JAMA.

[22] Wulf Hanson S., Abbafati C., Aerts J.G., Al-Aly Z., Ashbaugh C., Ballouz T., Blyuss O., Bobkova P., Bonsel G., Borzakova S. (2022). A global systematic analysis of the occurrence, severity, and recovery pattern of long COVID in 2020 and 2021. medRxiv.

[23] Donald J., Bilasy S.E., Yang C., El-Shamy A. (2024). Exploring the Complexities of Long COVID. Viruses.

[24] Tsampasian V., Elghazaly H., Chattopadhyay R., Debski M., Naing T.K.P., Garg P., Clark A., Ntatsaki E., Vassiliou V.S. (2023). Risk factors associated with post-COVID-19 condition: A systematic review and meta-analysis. JAMA Intern. Med..

[25] Garout M.A., Saleh S.A., Adly H.M., Abdulkhaliq A.A., Khafagy A.A., Abdeltawab M.R., Rabaan A.A., Rodriguez-Morales A.J., Al-Tawfiq J.A., Alandiyjany M.N. (2022). Post-COVID-19 syndrome: Assessment of short-and long-term post-recovery symptoms in recovered cases in Saudi Arabia. Infection.

[26] Alfadda A.A., Rafiullah M., Alkhowaiter M., Alotaibi N.M., Alzahrani M. (2022). Clinical and biochemical characteristics of people experiencing post-coronavirus disease 2019-related symptoms: A prospective follow-up investigation. Front. Med..

[27] Pérez Catalán I., Roig Martí C., Folgado Escudero S., Segura Fábrega A., Varea Villanueva M., Fabra Juana S., Domínguez Bajo E., Herrero Rodríguez G., Esteve Gimeno M.J., Palomo de la Sota D. (2024). Presence of COVID-19 self-reported symptoms at 12 months in patients discharged from hospital in 2020–2021: A Spanish cross-sectional study. Sci. Rep..

